# Explainable artificial intelligence in pancreatic cancer prediction: from transparency to clinical decision-making

**DOI:** 10.3389/fonc.2025.1720039

**Published:** 2025-12-17

**Authors:** Wardah Alharbi, Asma Abdullah Alfayez

**Affiliations:** 1 Department of AI and Bioinformatics, King Abdullah International Medical Research Centre (KAIMRC), Riyadh, Saudi Arabia; 2 King Saud Bin Abdulaziz University for Health Sciences (KSAU-HS), Riyadh, Saudi Arabia; 3 Ministry of National Guard - Health Affairs, Riyadh, Saudi Arabia

**Keywords:** precision oncology, pancreatic cancer, explainable artificial intelligence, machine learning, model interpretability, clinical decision support

## Abstract

**Background/Objectives:**

Pancreatic cancer (PC) remains among the most lethal malignancies worldwide, with a persistently low 5-year survival rate despite advances in systemic therapies and surgical innovation. Machine learning (ML) has emerged as a transformative tool for early detection, prognostic modelling, and treatment planning in PC, yet widespread clinical use is constrained by the “black box” nature of many models. Explainable artificial intelligence (XAI) offers a pathway to reconcile model accuracy with clinical trust, enabling transparent, reproducible, and clinically meaningful predictions.

**Methods:**

We reviewed literature from 2020–2025, focusing on ML-based studies in PC that incorporated or discussed XAI techniques. Methods were grouped by model architecture, data modality, and interpretability framework. We synthesized findings to evaluate the technical underpinnings, interpretability outcomes, and clinical relevance of XAI applications.

**Results:**

Across 21 studies on ML in PC, only three studies explicitly integrated XAI, primarily using SHAP and SurvSHAP. These methods helped identify key biomarkers, comorbidities, and survival predictors, while enhancing clinician trust. XAI approaches were categorized by staging (ante-hoc vs. *post-hoc*), compatibility (model-agnostic *vs*. model-specific), and scope (local *vs*. global explanations). Barriers to adoption included methodological instability, limited external validation, weak workflow integration, and lack of standardized evaluation.

**Conclusions:**

XAI has the potential to serve as a cornerstone for advancing transparent, trustworthy ML in PC prediction. By clarifying model reasoning, XAI enhances clinical interpretability and regulatory readiness. This review provides a technical and clinical synthesis of current XAI practices, positioning explainability as essential for translating ML innovations into actionable oncology tools.

## Introduction

Pancreatic cancer (PC) remains one of the most lethal malignancies worldwide, with a 5-year survival rate persistently below 10% despite advances in surgical techniques and systemic therapies (1–3) Globally, PC ranks as the 12th most common cancer, yet it is the third leading cause of cancer-related mortality in high-income countries such as Australia and the United States (4–6). In Saudi Arabia, PC represents a smaller but notable health burden (7). The incidence of PC has risen steeply in recent years, with reported new cases increasing from just 131 in 2005 to 579 in 2022 (8). Alarmingly, PC also carries the lowest five-year survival rate among all cancers in the Kingdom and is ranked as the eighth leading cause of cancer death (8, 9). Although its incidence remains lower compared to Western countries, the disease in Saudi patients often appears at advanced stages, reflecting global challenges of late detection and limited treatment options (8, 9). This disproportionate mortality burden underscores the urgent need for innovative approaches to improve early detection and patient outcomes.

The dismal prognosis of PC is primarily driven by late-stage diagnosis, aggressive tumour biology, and limited efficacy of available treatments (1, 2). Surgical resection combined with adjuvant chemotherapy remains the gold standard for long-term survival, but eligibility is generally restricted to patients with localised disease and good performance status (3, 6). Although neoadjuvant strategies and more radical surgical approaches have expanded resection possibilities to select stage III and even stage IV patients, the overall survival benefit remains modest, highlighting the necessity for earlier and more precise diagnosis (6).

Efforts to refine prognostic stratification have highlighted the potential of serum carbohydrate antigen 19-9 (CA19-9) as a biomarker for tailoring treatment intensity. Recent studies suggest that markedly elevated CA19–9 levels (>500 U/mL) in anatomically resectable PC may justify the use of intensive neoadjuvant chemotherapy (NAC), helping to identify subgroups more likely to benefit from systemic disease control and improved survival. However, the absence of standardised thresholds and inconsistent predictive performance of CA19–9 underscore a broader challenge: translating biomarkers into reliable, actionable tools for clinical decision-making (10).

No safe and effective population-level screening method currently exists for detecting PC at asymptomatic or early stages (11, 12). Conventional imaging modalities, including endoscopic ultrasonography (EUS), computed tomography (CT), magnetic resonance imaging (MRI), and positron emission tomography (PET), are hindered by high costs, limited sensitivity, and anatomical constraints associated with the pancreas’s retroperitoneal location (2, 3, 13–15). Consequently, microscopic premalignant lesions often evade detection, delaying diagnosis until advanced stages when therapeutic options are severely limited (2, 3).

The integration of computational intelligence into oncology has opened new avenues for overcoming diagnostic delays in PC (16, 17). Machine learning (ML), a core subset of artificial intelligence (AI), can leverage heterogeneous clinical, imaging, and molecular variables to detect patterns undetectable to human observation (18, 19). By incorporating demographic variables, patient history, laboratory markers, imaging features, and pathology findings, ML-based systems can support earlier diagnosis, improve risk stratification, and assist in treatment planning (20, 21). Unlike conventional rule-based diagnostics, ML can dynamically adapt to evolving datasets, potentially improving sensitivity and specificity in early disease diagnosis. This adaptability positions ML as a promising transformative technology to address one of the greatest challenges in PC care: late detection (2, 22). Despite promising advances, the real-world adoption of ML in PC remains limited by several challenges, including small and heterogeneous datasets, lack of external validation, and limited interpretability of model outputs. Clinical records are often incomplete or inconsistent, while imaging and biomarker datasets show substantial variability across institutions (2, 3, 22–24). Moreover, “black box” ML models, although accurate, frequently fail to provide clinicians with transparent explanations for their predictions, undermining trust and slowing regulatory approval. Given that even minor errors in oncology diagnostics can have serious consequences, ensuring model reliability, reproducibility, and interpretability is essential for safe clinical deployment (2, 23, 24).

Explainable artificial intelligence (XAI) offers a pathway to bridge the gap between high-performing models and clinical trust. XAI methods, such as Shapley Additive Explanations (SHAP), provide granular insights into model predictions by quantifying the relative contribution of individual features and aligning algorithmic reasoning with established clinical knowledge. Beyond supporting physician decision-making, XAI facilitates model refinement, bias detection, and regulatory compliance. In the context of PC, where treatment decisions often include complex trade-offs between surgical risk, chemotherapy tolerance, and disease progression, transparent AI systems hold the potential to advance precision and personalization of care (2, 23, 24). To our knowledge, this work is among the first technically rigorous review dedicated exclusively to the role of XAI in ML-based prediction of PC. By integrating perspectives from oncology, data science, and clinical informatics, this work provides a structured synthesis of XAI methods according to model architecture, data modality, and interpretability scope. The review critically appraises the clinical relevance, emphasising how enhanced transparency can strengthen oncologic decision-making and foster clinician trust. Furthermore, it identifies key translational barriers spanning methodological, operational, and regulatory domains, highlighting areas that require focused attention to enable the future integration of explainability into predictive pipelines. This work forms part of a dual-paper series, complemented by a companion article focused on feature engineering strategies and clinical integration of ML models for PC prediction, together intended to serve as a reference for advancing clinically actionable and trustworthy AI tools in pancreatic oncology.

## Scope, objectives, and review methodology

This article is the first in a two-part series examining ML applications for PC prediction. While the companion paper focuses on feature engineering and clinical integration, the present review is dedicated exclusively to XAI, a critical yet underdeveloped component of translational AI in oncology. The objective of this review is to provide a structured synthesis of XAI methods applied to ML-based PC prediction, classify these techniques according to model architecture, data modality, and interpretability domain, and critically evaluate their capacity to support oncological decision-making. By centering on explainability, this article highlights both the promise and current limitations of XAI in PC prediction, while identifying methodological and regulatory challenges that must be addressed to enable safe clinical deployment.

**Figure 1:**
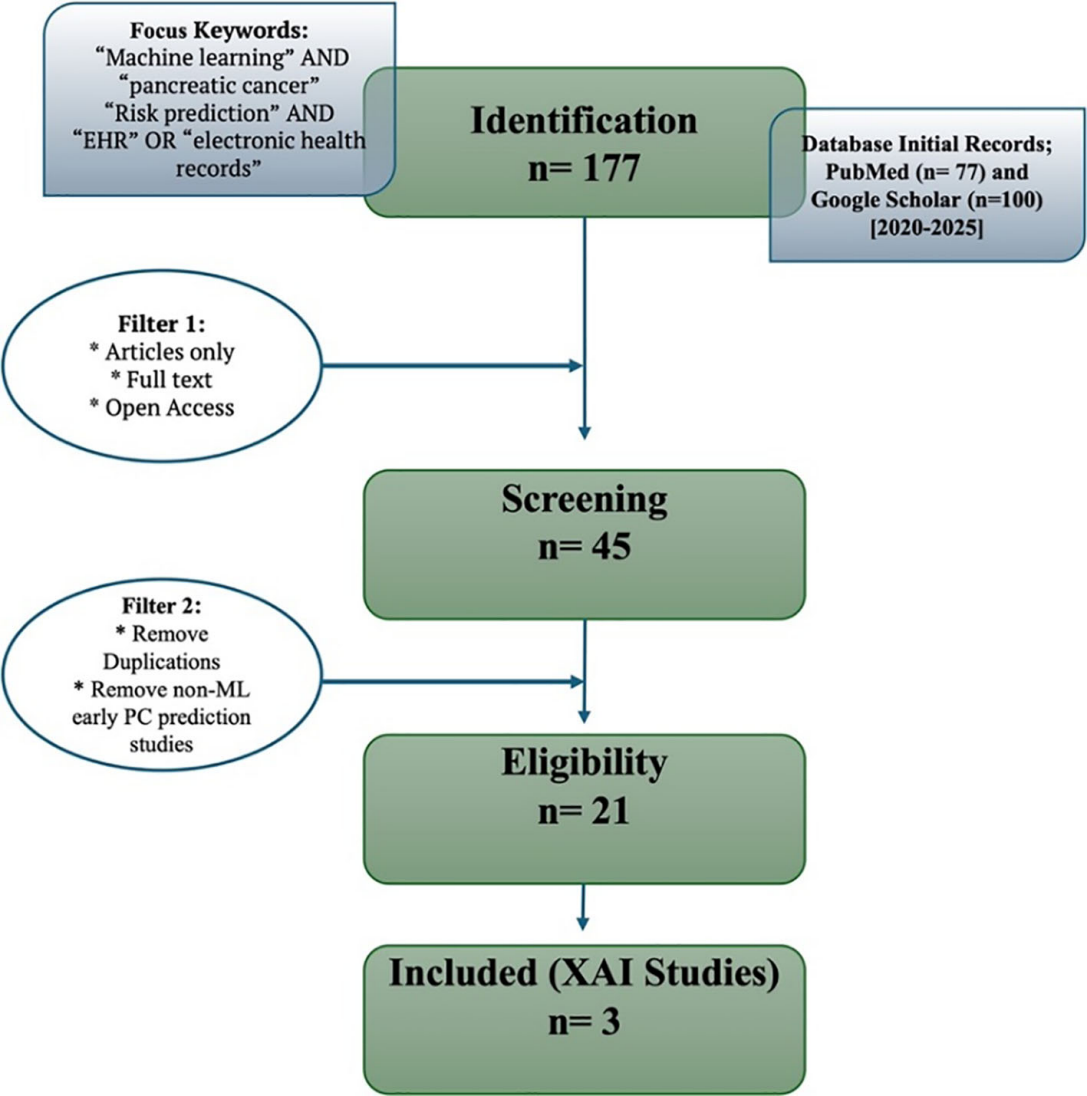
Flow diagram summarizing the literature selection process for ML studies in pancreatic cancer (2020–2025). From 177 initial records, 21 studies met the inclusion criteria, of which 3 incorporated XAI methods.

A focused literature search was conducted using PubMed as the primary database and Google Scholar for supplementary retrieval. Due to institutional access constraints, subscription-based databases such as Scopus and Web of Science were not included. Although this may limit exhaustive coverage, the resulting dataset reflects the major peer-reviewed studies published between 2020–2025 relevant to ML and XAI in PC prediction. The search strategy, filtering steps, and eligibility criteria are summarised in **Figure 1**.

**Figure 2:**
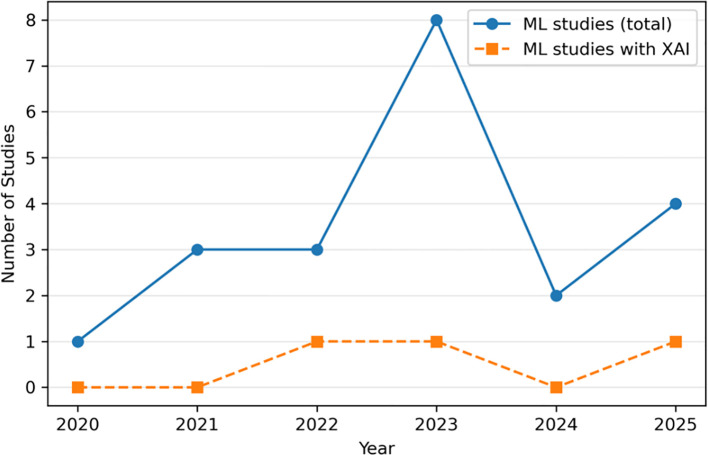
Temporal trend in ML and XAI studies for pancreatic cancer (2020–2025). Temporal trend (2020–2025) in machine-learning studies on pancreatic cancer and the smaller subset integrating XAI. Only 3 of 21 studies used XAI, despite a gradual increase in ML publications.

From an initial 177 records, removal of duplicates, restriction to full-text peer-reviewed articles, and screening for studies specifically addressing ML-based early prediction of PC resulted in 21 eligible ML studies, of which only three directly incorporated XAI methods. To contextualise this observation, we additionally present a temporal trend plot (**Figure 2**) illustrating the emergence of ML and XAI studies in PC from 2020 to 2025.

Taken together with its companion review, this work aims to serve as a comprehensive reference for researchers and clinicians developing interpretable, trustworthy, and clinically actionable ML models for PC.

## Explainability in practice: XAI applications

### Machine learning in oncology

ML has become a transformative force in oncology, providing data-driven solutions for early detection, prognostication, and treatment planning, particularly as the volume and complexity of medical data continue to expand. Broadly, ML methods are classified into supervised and unsupervised learning. Supervised learning algorithms, such as logistic regression (LR), support vector machines (SVM), random forests (RF), and gradient boosting (GB), use labelled datasets to predict known outcomes and are applied to both classification tasks (e.g., distinguishing malignant from benign lesions) and regression tasks (e.g., estimating survival probabilities or treatment responses). Unsupervised learning techniques, including principal component analysis (PCA), k-means clustering (KNN), Gaussian mixture models, density-based spatial clustering of applications with noise (DBSCAN), and balanced iterative reducing and clustering (BIRCH), operate on unlabelled data to uncover latent patterns, identify patient subgroups, and reduce dimensionality (22, 25–29). Deep learning (DL), a specialized branch of ML based on artificial neural networks (ANNs), expands these capabilities by using multi-layered architectures, such as convolutional and recurrent neural networks, to extract hierarchical representations from high-dimensional data. DL models have achieved state-of-the-art performance in oncology tasks such as tumour detection, segmentation, staging, mutation prediction, and radiomics-based analysis. Collectively, ML and DL have supported major advances in cancer susceptibility prediction, recurrence risk assessment, survival modelling, treatment toxicity forecasting, and genomic profiling (22, 25, 27, 30–32). These strengths are especially relevant to PC, where subtle early signals are often missed by conventional diagnostic pathways; by integrating demographics, comorbidities, biomarker trends, imaging features, and pathology findings, ML systems can identify high-risk individuals earlier and more consistently than rule-based approaches (1–34).

However, despite these advantages, several barriers limit the real-world application of ML in oncology, including heterogeneous and incomplete clinical datasets, limited external validation, challenges in multimodal data harmonisation, poor workflow integration, and the inherent opacity of many high-performing models (22, 24, 35, 36). These limitations underscore the urgent need for XAI, which can provide transparent, clinically aligned insights into model decision-making (1, 22, 23). The following sections therefore examine XAI frameworks most relevant to PC prediction and illustrate how explainability can bridge the gap between algorithmic performance and clinical usability.

### Overview of XAI methods

**Figure 3:**
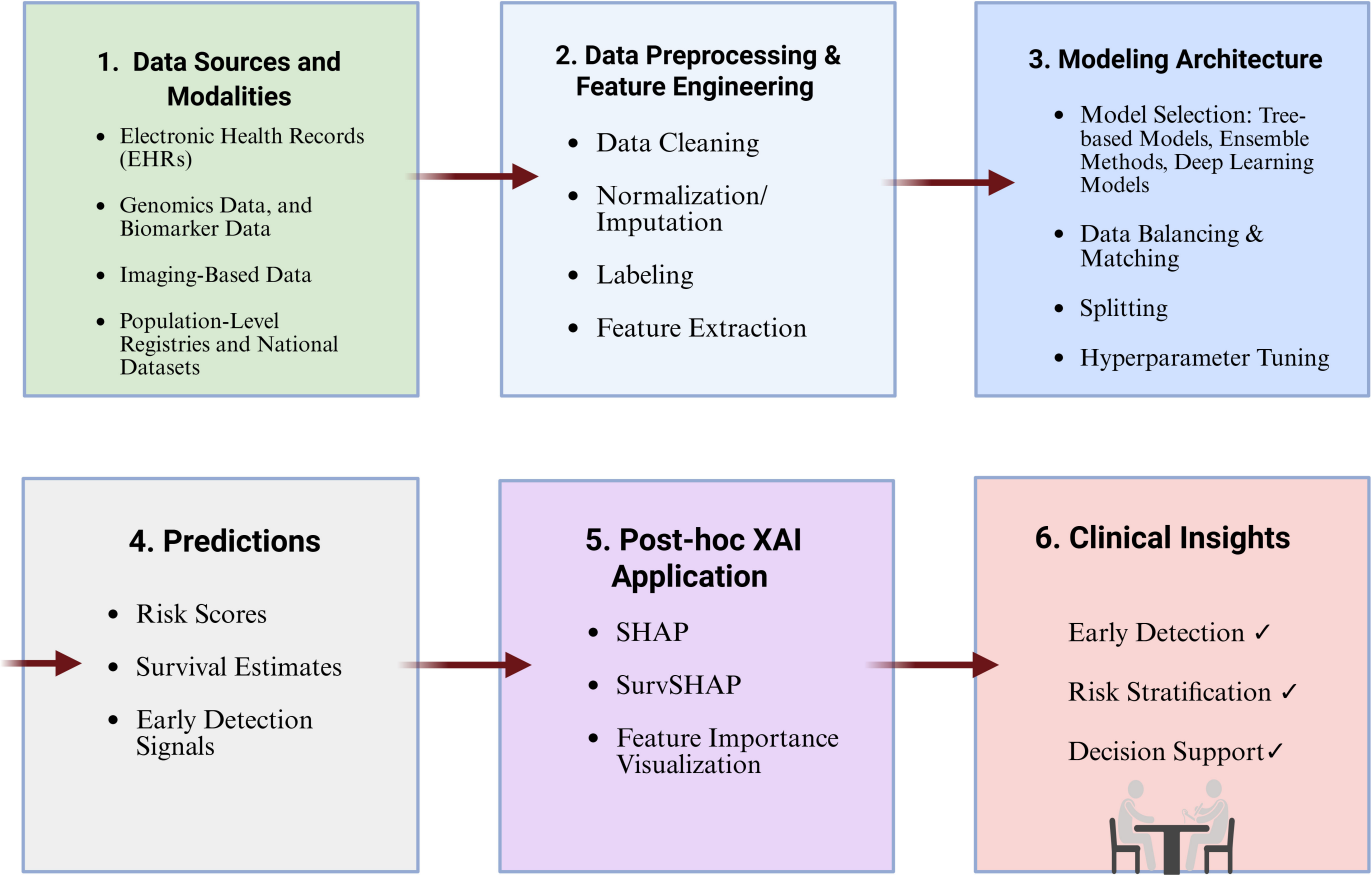
Overview of the XAI pipeline for pancreatic cancer prediction. Sequential workflow of an XAI-enhanced ML pipeline for PC prediction, from data sources and modalities, data preprocessing and feature engineering, modeling architecture to predictions, post-hoc XAI interpretation, and resulting clinical insights.

Building on this momentum, the integration of XAI into clinical ML systems has shifted from a complementary option to a fundamental requirement. As ML models are increasingly applied in diagnostics, prognostics, and risk stratification (1, 37), explainability becomes essential for ensuring transparency, reliability, and clinical accountability (37–39). To contextualize this evolution, **Figure 3** presents an overview of the XAI pipeline for PC prediction, summarizing how data sources, ML architectures, selected features and interpretability layers interact to yield clinically meaningful insights. In PC prediction, where decisions are high-stakes, clinicians must be able to trace and understand the rationale behind each risk score or treatment suggestion, whether derived from structured health records, imaging, or biomarker trends. Without such interpretability, even models with high predictive accuracy may be disregarded by healthcare professionals or encounter resistance from regulatory authorities (37–39).

To address these concerns, XAI introduces explanatory logic into the ML pipeline, enabling users to identify which input variables, such as biomarker levels, temporal disease sequences, or lab results, drive a given prediction. By enhancing transparency, XAI deepen model understanding, facilitates error analysis, supports fairness audits, and strengthens regulatory readiness. In complex clinical settings such as cancer care, where multidisciplinary collaborative decision-making is the norm, XAI provides each specialist, from radiologist to oncologist, with a rationale that can be interrogated and trusted (37–39). XAI methods are broadly categorised across three key dimensions that shape their operational behaviour and clinical suitability. The first dimension, staging, differentiates between ante-hoc and *post-hoc* methods. Ante-hoc (or intrinsic) explainability method is embedded directly within the model architecture itself, such as decision trees or rule-based models, making the model inherently interpretable. In contrast, *post-hoc* methods are applied after model training, serving to approximate or uncover the reasoning behind predictions from complex, dense architectures such as deep neural networks (DNNs). The second dimension, model compatibility, assesses whether an explanation method is model-agnostic, meaning it can be applied to any ML algorithm regardless of structure (e.g., Local Interpretable Model-agnostic Explanations (LIME) or SHAP), or model-specific, tailored to specific architectures such as CNNs (e.g., Gradient-weighted Class Activation Mapping (GradCAM)). The third dimension relates to the scope of explanation, differentiates between local methods, which provide interpretability for individual predictions and are critical for case-by-case clinical decision-making, and global methods, which explain and characterise the overall behaviour of the model, offering insights into feature importance and decision trends across the dataset (37, 39, 40). **Table 1** outlines a comparative landscape of widely used XAI techniques, highlighting their classification across these dimensions, their technical underpinnings, and representative healthcare applications. Understanding this framework is essential for choosing an appropriate XAI strategy tailored to the clinical task at hand, ensuring that the resulting model is not only accurate but also transparent, justifiable, defensible, and aligned with the expectations of clinicians, patients, and regulators (39).

**Table 1:** Overview of widely used XAI techniques: staging, scope, and clinical use cases.

AIX method	Description	Stage-based classification	Compatible ML models	Scoping	Example of healthcare applications
Shapley Additive Explanations (SHAP)	Uses game theory to attribute importance to each feature by computing Shapley values	*Post-hoc*	Model-agnostic	Local and Global	Predicting risk of PC using EHR data, and identifying how features like HbA1c or BMI influence individual predictions (2, 41, 42)
Local Interpretable Model-agnostic Explanations (LIME)	Builds simple surrogate models locally around each prediction	*Post-hoc*	Model-agnostic	Local	Which lab results (e.g., lactate, WBC count) contributed most to the disease (39)
Partial Dependence Plots (PDP)	Shows the average effect of a feature on model predictions	*Post-hoc*	Model-agnostic	Global	Visualising how variations in glucose or age affect the predicted probability of diabetes complications or cancer risk (39)
Gradient-weighted Class Activation Mapping (GradCAM)	Highlights image regions relevant to CNN-based predictions using gradient information	*Post-hoc*	Model-Specific Explanations, CNNs, vision-based DL models	Local	Highlights the most influential regions in radiology scans (CT, MRI, X-rays) (39, 40)
DeepLIFT (Deep Learning Important FeaTures)	Traces contributions of neurons by comparing activations to a reference baseline	*Post-hoc*	Model-Specific Explanations, DNNs especially feedforward and genomic networks	Local	Used to interpret gene expression models, HER-based DL models, and image classification tasks (e.g., tumour localization or subtype classification) (39)

Among the widely adopted XAI methods, SHAP has emerged as a reference standard for clinical model interpretation, offering both local and global explanations through additive feature attributions grounded in cooperative game theory. While SHAP values are inherently local, quantifying the contribution of each feature to an individual prediction, they can be aggregated across patients to yield global insights into model behaviour. Aggregated SHAP summaries, such as mean absolute SHAP values or global importance plots, enable population-level interpretability and highlight consistently influential predictors across the cohort (39–41). For example, a large-scale oncological study utilised aggregated SHAP values to identify global biomarker patterns influencing colorectal-cancer risk. More recently, Almisned et al. (2), applied SHAP in an ensemble ML framework for early detection of PC; by presenting SHAP-based global feature rankings alongside patient-level attributions, the authors demonstrated how SHAP can inform both individual clinical decisions and cohort-wide predictor assessment. These studies illustrate how SHAP transcends strictly local explanation to enable global model interpretability in clinical-AI applications (37, 39).

LIME, although less stable, generates local surrogate models around specific predictions and its broadly model-agnostic applicability. For imaging applications, GradCAM highlights key regions within radiological scans that drive classifications, supporting visual diagnostic interpretation. PDPs provide global insights into the marginal effect of individual features but are limited by their assumption of feature independence and vulnerability to feature correlation. DeepLIFT, optimised for deep architectures and networks, delivers efficient layer-wise attributions by comparing neuron activations, and has proven useful in complex biomedical domains such as genomics (39–41).In practice, *post-hoc* approaches dominate clinical applications due to their flexibility; they can be applied without retraining the models and generate outputs (e.g., feature rankings, heatmaps, or rule sets) that align with clinical reasoning. However, choosing the right method remains nontrivial; as techniques and approaches vary in assumptions, stability, and interpretive depth. While some methods are inherently interpretable (ante-hoc), others retrospectively approximate explanation (*post-hoc*); some offer patient-level insights (local), while others describe model-wide behaviour (global) (37, 39).

This section aimed to equip researchers and practitioners with a foundational understanding of XAI tools most applicable to PC prediction. Clarifying these distinctions enhances transparency, supports responsible deployment, and ultimately bridges the gap between ML innovations and actionable clinical insight.

### Studies utilising XAI in PC prediction

XAI has become increasingly critical in healthcare, particularly for high-stakes decisions such as cancer diagnosis and prognosis. In our structured review of 21 studies on PC prediction, only three explicitly applied XAI methods (2, 41, 42), with one further study relying on feature importance analysis of ICD codes (33). These XAI applications, principally SHAP and SurvSHAP, illustrate how interpretability can strengthen clinical trust and yield actionable insights (2, 41, 42). A detailed comparison of the three studies that integrated XAI into ML-based PC prediction is provided in **Table 2**. A full overview of all 21 studies is provided in **Supplementary Table S1**, with details on model type, data source, and feature design. Here, however, we focus on the three studies that directly integrated XAI, while feature-engineering-centred works, clinical variables, and integration into care pathways are addressed in a forthcoming companion review. Taken together, the two papers offer a comprehensive assessment of how ML can be made both interpretable and clinically actionable in the context of PC.

**Table 2:** Studies integrating XAI methods in pancreatic cancer (PC) prediction.

Study	Population/Dataset	Model(s)	Features	XAI method	Key findings/clinical insights
Almisned et al. (2025) (2)	Early PC cases; clinical and biomarker dataset	Six machine learning (ML) classifiers + Ensemble voting	Clinical variables + biomarkers (e.g., TFF1, LYVE1)	SHAP	SHAP identified benign sample diagnosis, TFF1, and LYVE1 as top predictors. Provided interpretable thresholds; enabled patient-level risk profiling and tailored treatment planning.
Keyl et al. (2022) (42)	203 patients with advanced pancreatic ductal adenocarcinoma (PDAC)	Random Survival Forest (RSF)	carbohydrate antigen 19-9 (CA19-9), C-reactive protein (CRP), neutrophil-to-lymphocyte ratio (NLR), age, metastatic status	SHAP	CRP and NLR emerged as dominant predictors of poor survival; higher serum proteins and M0 status linked to improved outcomes. SHAP improved transparency of survival modelling and guided prognostic reasoning.
Chen et al. (2023) (41)	EHR data from two US health systems	RSF, eXtreme gradient boosting (XGB), Cox regression	Demographics, clinical history, abdominal pain	SurvSHAP	Age most influential predictor across models. SurvSHAP revealed heterogeneous predictive logic across algorithms, enhancing trust and informing targeted screening strategies.

In a recent study by Almisned et al. (2), researchers proposed an ensemble ML framework for early PC detection using clinical and biomarker features. Six ML algorithms were evaluated alongside an ensemble voting classifier, with SHAP applied for model interpretation. SHAP was used to quantify and visualise the contribution of each feature to the model’s predictions. The top predictive variables identified by SHAP included benign sample diagnosis, TFF1, and LYVE1 as top predictors with strong positive influence on early-stage PC prediction. Notably, SHAP suggested interpretable thresholds and feature relevance that clinicians could interpret and validate. For instance, elevated LYVE1 levels were consistently associated with malignancy, suggesting the need for targeted interventions such as non-invasive imaging or liquid biopsies. Furthermore, SHAP also enabled patient-level risk assessments, reinforcing its value for multidisciplinary decision-making in designing tailored treatment plans. The study emphasised that model interpretability is not merely vital for regulatory transparency but also for clinical adoption, reinforcing XAI’s function in developing trust and informed decision-making in oncology settings (2).

Another study, Keyl et al. (42), examined the application of SHAP within a random survival forest (RSF) model trained on clinical data from 203 patients with advanced pancreatic ductal adenocarcinoma (PDAC). Baseline predictors included CA19-9, C-reactive protein (CRP), neutrophil-to-lymphocyte ratio (NLR), age, and metastatic status. SHAP analysis revealed CRP and NLR as the dominant and most influential predictors of poor survival, followed by age and CA19-9. In contrast, higher serum protein levels and absence of metastasis disease (M0 status) were associated with improved survival outcomes. SHAP enabled transparent visualisation of each variable’s directional influence on survival predictions, enhancing clinician understanding and guiding more nuanced prognostic evaluations. This study validates the potential of XAI not just in classification tasks but also in survival modelling critical to advanced-stage cancer management (42).

SurvSHAP visualisations uncovered heterogeneous predictive logic across models, increasing transparency and confidence in outputs, with implications for targeted screening strategies.

In an effort to improve early detection of sporadic PC, another study by Chen et al. (41) compared the predictive performance of RSF, eXtreme gradient boosting (XGB), and Cox proportional hazards regression models using electronic health record (EHR) data from two main US healthcare systems. The study incorporated SurvSHAP, an XAI method specifically tailored for survival models, to explain feature contribution across algorithms. Age emerged as the most influential predictor across all three models, while abdominal pain contributed minimally in the RSF and XGB models but was more prominent in Cox regression model. SurvSHAP visualisations uncovered heterogeneous predictive logic across models, increasing transparency and confidence in model outputs, with implications for targeted screening strategies (41).

Despite the proliferation of ML-based approaches in PC prediction, the adoption and integration of XAI remains limited. The few studies that integrated XAI, primarily through SHAP and its survival adaptation SurvSHAP, demonstrated how interpretability can enhance model transparency, clinical trust, and actionability. These studies reflect the growing recognition that interpretability is not optional but a core necessity for translating AI solutions into meaningful clinical practice (2, 41, 42).

### Interpretability and explainability in clinical AI: conceptual boundaries, cross-domain perspectives, and relevance to pancreatic cancer

The distinction between interpretability and explainability is fundamental to evaluating the transparency, safety, and trustworthiness of AI systems in high-stakes healthcare applications. Although these terms are often used interchangeably across the XAI literature, leading scholars have emphasized that they embody distinct, and sometimes competing, objectives (38, 43–46). We explicitly align our definitions with established frameworks while acknowledging that different fields conceptualize these terms differently. Our synthesis therefore represents both a consolidation of prior literature and a clinical contextualization tailored to PC prediction.

*Interpretability* refers to models whose internal structure is directly understandable by humans without requiring external explanatory tools. Interpretability is achieved through domain-aligned constraints, such as sparsity, rule-based logic, monotonicity, or additive structure, that make predictions transparent and traceable. Examples include LR with clinically meaningful coefficients, decision trees, Certifiably Optimal Rule Lists (CORELS), and generalized additive models (GAMs) (43–45, 47). In oncology, such models enable clinicians to confirm that predictors align with biological and epidemiological knowledge, e.g., elevated CA19–9 or rapid weight loss increasing PC risk, thereby supporting real-time auditing, error detection, and the integration of contextual cues absent from EHR data (10). This advantage parallels findings in other high-stakes fields, including criminal justice, materials engineering, and air-quality forecasting, where interpretable models have repeatedly prevented bias, confounding, and model misuse (43).

*Explainability*, in contrast, refers to *post hoc* analytic techniques that attempt to summarize or approximate the behaviour of complex, non-transparent (“black-box”) models after training. Methods such as SHAP, SurvSHAP, LIME, Grad-CAM, Anchors, and Partial Dependence Plots (PDP) generate feature-level or instance-level explanations without exposing the model’s internal computations (45, 47). Some of these tools are widely used in PC prediction because high-performing models, such as XGBoost, random forests, or DNNs, often sacrifice inherent transparency to maximize predictive accuracy (2, 41, 48). However, as Rudin (43) argues, *post hoc* explanations cannot fully replicate a black-box model’s reasoning; if an explanation perfectly captured the model, the model itself would be unnecessary (43, 44). This fidelity gap is particularly consequential in medicine, where misaligned or incomplete explanations can mislead clinicians, obscure confounding, or mask algorithmic biases.

**Figure 4:**
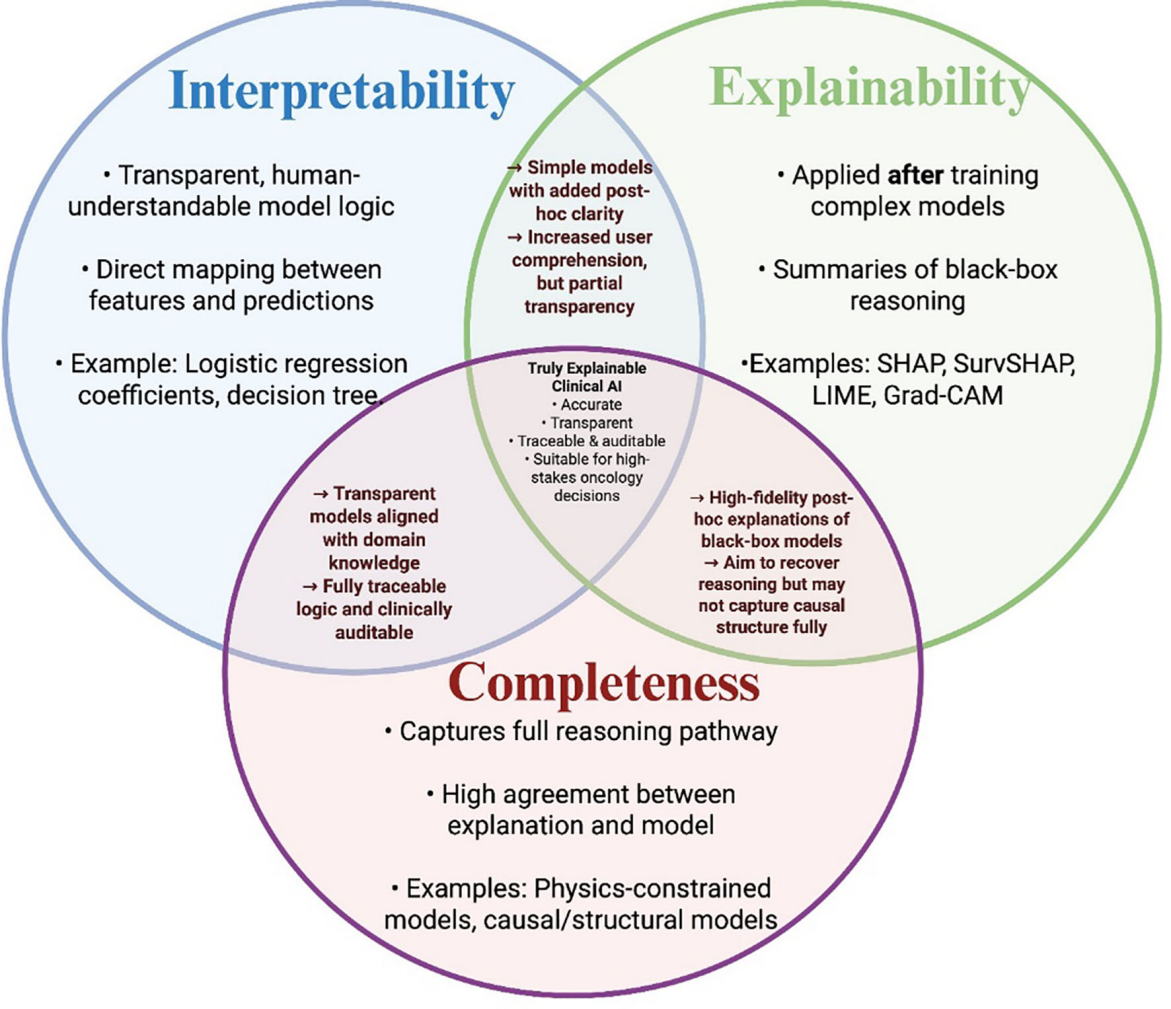
Interpretability, explainability, and completeness in clinical AI.

To reconcile these perspectives, we present interpretability, explainability, and completeness as overlapping but non-identical constructs (**Figure 4**). *Interpretability* focuses on human comprehension of the model’s inherent logic. *Explainability* provides approximations of the reasoning process for otherwise opaque models. *Completeness* describes the degree to which either approach faithfully captures the true computational or causal pathways (49, 50).

This conceptual model aligns with the XAI distinctions articulated (43), recent engineering literature (45), and foundational ML theory (44), while acknowledging that our synthesis is tailored to clinical decision-making.

Cross-domain comparisons also illustrate that the stakes differ by discipline. In materials science and nanoporous materials modelling, for example, SHAP is routinely used as a global and local *post hoc* tool to analyse physics-related feature importance despite inherent limitations in capturing mechanistic pathways (37, 38, 45). In contrast, medical domains, where decisions directly affect patient outcomes, emphasize accountability, causal faithfulness, fairness, and regulatory acceptance (37, 38). This divergence reinforces the argument that explainability alone may be insufficient for clinical deployment, yet practically necessary when high-performing black-box models are unavoidable.

In PC, these distinctions have concrete implications. Interpretable models allow clinicians to validate risk factors, temporal disease signatures, and biomarker behaviour, strengthening trust and facilitating uptake into clinical pathways. Explainability tools, such as SHAP and SurvSHAP, allow high-accuracy black-box models to be interrogated for biological plausibility, temporal trends, and subgroup bias, critical for early detection where false reassurance or misinterpretation carries significant risk. Together, these perspectives highlight that a hybrid strategy, prioritizing interpretable models when feasible and applying rigorous, high-fidelity *post hoc* XAI when necessary, provides the most robust pathway toward clinically actionable and ethically sound AI for PC prediction.

### Gaps in interpretability and physician trust

While ML holds considerable promise for healthcare, its integration into clinical practice remains constrained by persistent challenges in interpretability and physician trust. Interpretability is not optional; it is foundational for responsible adoption. Yet many high-performing models often operate as black boxes, composed of deeply layered, nonlinear structures with millions of parameters, making their decision processes inherently opaque. Even with the aid of XAI tools, explanations often remain partial: attribution heatmaps may highlight influential features without explaining their clinical significance. For frontline clinicians lacking advanced ML or statistical training, these explanations may remain inaccessible or insufficiently actionable, widening the gap between developers and end-users (37–40).

Trust is further undermined by the inconsistency of some XAI methods, whose explanations can be unstable or misleading. Several studies question the reliability of *post-hoc* explanations, citing their susceptibility to instability or misrepresentation. The lack of standardised metrics for assessing explanation quality worsens this issue, limiting comparison across clinical contexts (37, 38).

At the system level, barriers such as limited infrastructure, poor EHR integration, non-intuitive interfaces, and shifting regulatory standards further erode clinician confidence in AI-driven recommendations, particularly in time-sensitive scenarios (37–39).

Nonetheless, XAI remains central to fostering trust. Techniques such as SHAP and LIME can help translate complex predictions into clinically interpretable elements, enabling clinicians to question, validate, or contextualise predictions based on patient-specific data. This transparency reframes the clinician–AI relationship from one of scepticism to collaboration. Bridging the gap between interpretability and physician trust will, however, demand more than technical advances. It essentially requires aligning XAI outcomes with clinical reasoning, simplifying user interaction, and developing rigorous evaluation standards. Only through such multidimensional progress can AI move beyond theoretical promise and become a trusted tool in routine care (37–39).

## Discussion and future directions

The integration of XAI into ML-based prediction of PC represents a pivotal opportunity to bridge the gap between computational innovation and clinical translation. While conventional models achieve strong predictive performance, their opacity undermines physician trust, regulatory approval, and ultimately, real-world clinical deployment (2, 23, 24). Our synthesis highlights that XAI is increasingly regarded as a foundational requirement for precision oncology. However, the review also uncovers limitations that must be addressed to achieve clinical maturity.

First, the adoption of XAI in PC prediction is limited, with only a handful of studies explicitly incorporating interpretability frameworks such as SHAP or SurvSHAP (2, 41, 42). Even within these applications, explanations are typically restricted to surface-level feature attributions, rarely aligned with pathophysiological knowledge. Bridging this gap requires moving beyond feature rankings to clinically grounded narratives that link model predictions to biological plausibility and treatment relevance.

Second, methodological fragility is pressing concern. Many *post-hoc* techniques are unstable, with outputs sensitive to minor data perturbations, undermining reproducibility. Developing standardised benchmarks for explanation quality, analogous to accuracy or area under the curve (AUC) metrics for predictive performance, is essential for robust clinical evaluation (23, 37).

Future work should also prioritise scalability and external validation. Current studies are largely confined to single-institution cohorts or limited datasets, which restricts generalisability. Cross-institutional federated learning frameworks, combined with XAI, may strengthen robustness while preserving data privacy (22, 24, 35). Furthermore, integration into EHR systems and clinical workflows is similarly underdeveloped. Effective deployment will require intuitive, clinician-facing interfaces where model explanations are seamlessly presented alongside conventional diagnostic tools. Such integration must align with evolving regulatory standards that increasingly demand transparency in algorithmic decision-making (37, 38, 41, 51).

Multimodal explainability also demands attention. PC prediction increasingly involves EHR variables, biomarkers, imaging, and genomics. Future work should aim to unify explanations across modalities, enabling a holistic understanding of patient-level insights and supporting collaborative decision-making in multidisciplinary oncology boards (33, 52, 53).

Finally, physician trust must remain central to future directions. Technical innovation in explainability will have limited impact unless it resonates with clinician reasoning and patient communication. Human-centred design, co-development with end-users, and iterative feedback between data scientists and healthcare providers will be indispensable for aligning XAI with clinical decision-making (2, 37, 38, 41, 51). Such multidimensional approach, blending methodological rigour, clinical grounding, and human-centric design, will be essential if explainable ML is to move from proof-of-concept to a transformative tool in PC care.

## Conclusion

This review establishes explainability as a cornerstone for translating ML innovations into clinically actionable tools for PC prediction. By appraising XAI methods, we demonstrate their potential to enhance transparency, refine prognostic reasoning, and build clinician trust, while also outlining the barriers that currently hinder their adoption. The limited number of studies explicitly applying XAI underscores both the novelty of this domain and the urgency of advancing it. Future research must prioritise methodological robustness, multimodal integration, external validation, and user-centred deployment to unlock the full clinical potential of explainable ML.

As the first article in a two-part series, this review defines the technical and clinical landscape of XAI in PC prediction, while its companion paper addresses feature engineering and integration strategies. Together, they provide a comprehensive roadmap for developing interpretable, trustworthy, and impactful AI solutions in oncology.

In parallel, we are conducting an IRB-approved study (NRR25/67/3, KAIMRC) that aims to develop an ML-based model for predicting the risk of PC in patients with chronic metabolic disorders, using data from local EHRs. The ultimate aim of this project is to support early diagnosis of PC and targeted surveillance, improving patient outcomes. Importantly, the integration of XAI will ensure that this predictive framework remains interpretable and clinically trustworthy, directly addressing the translational gaps highlighted in this review.

## Supplementary material

Supplementary Table 1 Structured summary of included studies.
